# A Pilot Study Characterizing Flow Patterns in the Thoracic Aorta of Patients With Connective Tissue Disease: Comparison to Age- and Gender-Matched Controls *via* Fluid Structure Interaction

**DOI:** 10.3389/fped.2022.772142

**Published:** 2022-05-04

**Authors:** Joseph A. Camarda, Ronak J. Dholakia, Hongfeng Wang, Margaret M. Samyn, Joseph R. Cava, John F. LaDisa

**Affiliations:** ^1^Department of Pediatrics, Division of Cardiology, Herma Heart Institute, Children's Wisconsin and the Medical College of Wisconsin, Milwaukee, WI, United States; ^2^Department of Biomedical Engineering, Marquette University the Medical College of Wisconsin, Milwaukee, WI, United States; ^3^Departments of Medicine, Division of Cardiovascular Medicine and Physiology, Medical College of Wisconsin, Milwaukee, WI, United States

**Keywords:** Marfan syndrome, computational modeling, patient-specific modeling, wall shear stress, Loeys-Dietz syndrome

## Abstract

Prior computational and imaging studies described changes in flow patterns for patients with Marfan syndrome, but studies are lacking for related populations. This pilot study addresses this void by characterizing wall shear stress (WSS) indices for patients with Loeys-Dietz and undifferentiated connective tissue diseases. Using aortic valve-based velocity profiles from magnetic resonance imaging as input to patient-specific fluid structure interaction (FSI) models, we determined local flow patterns throughout the aorta for four patients with various connective tissue diseases (Loeys-Dietz with the native aorta, connective tissue disease of unclear etiology with native aorta in female and male patients, and an untreated patient with Marfan syndrome, as well as twin patients with Marfan syndrome who underwent valve-sparing root replacement). FSI simulations used physiological boundary conditions and material properties to replicate available measurements. Time-averaged WSS (TAWSS) and oscillatory shear index (OSI) results are presented with localized comparison to age- and gender-matched control participants. Ascending aortic dimensions were greater in almost all patients with connective tissue diseases relative to their respective control. Differences in TAWSS and OSI were driven by local morphological differences and cardiac output. For example, the model for one twin had a more pronounced proximal descending aorta in the vicinity of the ductus ligamentum that impacted WSS indices relative to the other. We are optimistic that the results of this study can serve as a foundation for larger future studies on the connective tissue disorders presented in this article.

## Introduction

Blood flow patterns in the aortic arch and descending thoracic aorta (dAo) are unique when compared with other portions of the arterial vasculature. For example, helical flow is present within the aortic arch under normal conditions and is thought to influence flow patterns at the origins of the carotid and subclavian arteries (1). Characterizing any deviations from these normal flow patterns may be important for optimal operative interventions involving the thoracic aorta with the goal of decreasing the likelihood of later, long-term aortic pathology. For instance, transient periods of turbulence during systole (due to modest differences in local vessel geometry from thoracic aortic diseases or surgery) could cause downstream flow disturbances. Such disturbances have been associated with local dilation (2, 3) and can subsequently impact indices of wall shear stress (WSS; defined as the tangential force per unit area exerted on a vessel wall as a result of flowing blood). Abnormal WSS has been related to pathology in the thoracic aorta. In a study of 10 middle-aged adults with preexisting plaques, areas of low time-averaged WSS (TAWSS) were found in a rotating pattern progressing down the dAo and correlated with areas of atherosclerosis (4). Excessively high WSS can also be deleterious by initiating platelet aggregation (5).

Marfan syndrome is one type of connective tissue disorder that impacts several tissues/organs, including the cardiovascular system, where it can lead to thoracic aortic aneurysms (6). Prior research indicates that thoracic aortic aneurysms are the leading cause of death for patients with Marfan syndrome (7). Despite the uniqueness and importance of flow patterns in the thoracic aorta, there are few studies characterizing local blood flow patterns for connective tissue diseases beyond Marfan syndrome (8). To date, most computational studies of patients with Marfan syndrome have employed rigid computational fluid dynamics (CFD) models, which have the potential to reveal detailed spatiotemporal quantification of hemodynamic indices, including WSS, based on magnetic resonance imaging (MRI) and blood pressure (BP) data (9–19). A recent report quantified several indices by CFD, including WSS, for patients with Marfan syndrome before and after surgery to implement personalized external aortic root support (20). Some local differences were noted after surgery, but values were largely similar. This investigation builds from this prior work by conducting fluid structure interaction (FSI) modeling that includes local wall deformation of the aorta for patients with Loeys-Dietz, connective tissue disease of unclear etiology, and native (i.e., untreated) Marfan syndrome, as well as fraternal (i.e., dichorionic) twin patients with Marfan syndrome who underwent valve sparing root repair. All results are interpreted relative to those from age- and gender-matched control patients. The approaches employed for this study assigned local tissue properties as well as physiological inflow profiles and outlet boundary conditions to replicate clinical measurements. With these advancements, we aim to more accurately replicate the physiological conditions for patients with these connective tissue anomalies, and therefore help to further reveal differences in WSS and related indices vs. control participants. Thus, this pilot investigation may aid future long-term studies of morbidity related to aortic vascular disease.

## Methods

### Magnetic Resonance Imaging

Following Children's Wisconsin Institutional Review Board approval, MRI was performed for patients with connective tissue anomalies. MRI data from control participants with ages, genders, and Reynolds numbers aiming to closely match these patients were also obtained. Prior to protocol enrollment, verbal and written information was provided, and informed consent was obtained from participants. Details of the patients and control participants are shown in Table 1 (10 men and two women aged 18–55 years). Patients had various connective tissue diseases (Loeys-Dietz with native aorta; connective tissue disease of unclear etiology with native aorta in female and male patients; and an untreated patient with Marfan syndrome). All imaging was conducted as part of clinically ordered sessions or ongoing research.

**Table 1:** Hemodynamic indices, diagnosis, and aortic dimensions.

**Connective tissue disorder**	**Age**	**Gender**	**Diagnosis**	**Operation**	**Cardiac index (L/min/m^**2**^)**	**AscAo diameter (mm)**	**dAo diameter (mm)**	**AscAo/dAo diameter ratio**	**Difference in AscAo/dAo diameter vs. control (%)**	**Reynolds number (dimension- less)**
Loeys Dietz	55	M	Genetic-TGFBR 2 mutation	n/a	3.0	35.0	21.4	1.63	29.3	923
Control	57	M	-	-	2.5	32.2	25.5	1.26	-	910
										
Unknown etiology-male	38	M	Phenotypic diagnosis; Genetic-negative for FBN-1, TGFBR1, TGFBR 2	n/a	3.1	28.9	19.0	1.52	17.4	1,380
Control	32	M	-	-	4.5	26.9	20.7	1.30	-	1,520
Unknown etiology-female	24	F	Genetic-negative for FBN1, FBN1 del, TGFBR1, TGFBR2, MYLK, MYH11, ACTA 2	n/a	3.2	32.9	18.1	1.82	20.7	1,130
Control	23	F	-	-	2.0	22.0	14.6	1.51	-	823
										
Marfan syndrome–twin A	22	M	Genetic-FBN1 exon 30 mutation	Valve sparing root replacement	2.9	28.0	17.9	1.56	24.0	1,190
Control	26	M	-	-	2.5	26.3	20.9	1.26	-	1,190
Marfan syndrome–twin B	22	M	Genetic-FBN1 exon 30 mutation	Valve sparing root replacement	3.3	26.8	16.2	1.65	13.5	1,350
Control	24	M	-	-	3.5	28.8	19.8	1.46	-	1,550
Marfan syndrome-native	18	M	Phenotypic diagnosis	n/a	6.0	22.8	16.2	1.41	10.6	2,710
Control	18	M	-	-	3.2	25.1	19.7	1.28	-	1,520

*Reynolds number calculations assume a blood density of 1.06 g/cm^3^ and dynamic viscosity of 4 cP*.

Gadolinium-enhanced (0.4 ml/kg; gadodiamide, Omniscan®, GE Healthcare, Waukesha, WI, USA) MR angiography (MRA) was performed with a breath-held 3D fast gradient echo sequence using a 1.5T Symphony® scanner (Siemens Healthcare, Erlangen, Germany). Slice thickness was 2.0 mm, with 56–60 sagittal slices per volume. A 384 × 192 acquisition matrix (reconstructed to 384 × 256) was used with a field of view (FoV) of 25 × 42 cm^2^ (spatial resolution of 0.65 × 1.64 mm). Other parameters included a repetition time (TR) of 4.3 ms, echo time (TE) of 1.4 ms, and a flip angle of 25°.

Time-resolved, velocity-encoded two-dimensional anatomic and through-plane phase-contrast MRI (PC-MRI) was performed orthogonally in the ascending aorta (AscAo) near the main pulmonary artery, in the dAo at the level of the diaphragm, and orthogonal to the arch origins of the head and neck vessels. An additional PC-MRI scan was obtained through the aortic valve for all patients, and when possible for control participants. The data were used to create spatiotemporally varying computational model inlets delineated by the patient's aortic valve as previously described in detail (21, 22) and briefly summarized in the boundary conditions section below. Heart rates ranged from 82 to 92 bpm (with R-R ranging from 652 to 732 ms); 25 images were reconstructed for the average R-R interval. Imaging parameters included TR, TE, and flip angle of 46 ms, 3.8 ms, and 30°, respectively. The FoV was 30 × 22.5 cm^2^ with an acquisition matrix of 256 × 192 and a slice thickness of 7 mm, resulting in a voxel size of 1.17 mm × 1.17 mm × 7 mm. Subjects breathed freely during PC-MRI acquisition, and data were retrospectively gated to the cardiac cycle. After scanning, supine, bilateral upper and lower extremity BP assessment was performed using a Dinamap BP system (GE Healthcare, Waukesha, WI, USA). Cardiac indexes, aortic dimensions, and mean Reynolds numbers are provided in Table 1. Dimensions are taken from the computational models created using MRA data according to the details below, which we understand to generally be a diastolic representation of vessel morphology.

### Computational Model Construction

Computational versions of the aorta and arteries of the head and neck were created from MRA imaging data using Simvascular (Simtk.org) software as discussed previously (15). Models were discretized using a commercially available, automatic mesh generation program (MeshSim, Simmetrix, Clifton Park, NY, USA). Meshes contained ~4 million tetrahedral elements, and localized refinement was performed until results were independent of the mesh as discussed elsewhere (12) using an adaptive technique (23, 24) to deposit more elements near the luminal surface and in anatomical regions prone to flow disruption (14).

### Inlet Boundary Conditions

PC-MRI data were used to calculate time-resolved volumetric blood flow as previously described (16, 25). A time-varying plug flow inlet based on the measured AscAo flow was created, but with a restricted cross section determined from time-varying PC-MRI magnitude data at the level of the valve (21). A normal trileaflet valve was assumed for control participants and confirmed using the imaging data mentioned above, when possible.

### Outlet Boundary Conditions and Wall Deformation

Flow from the innominate, left common carotid artery, left subclavian artery, and dAo were used together with BP data to prescribe physiological outflow boundary conditions using three-element Windkessel approximations (26). The three-element Windkessel accounts for vessels distal to computational model branches using three parameters with physiological meaning, namely, characteristic resistance (R_c_), capacitance (C), and distal resistance (R_d_). The total arterial capacitance (TAC) for each patient was determined from inflow and BP data, assuming a characteristic to total resistance ratio of 6% (27). The TAC was then distributed among outlets according to their blood flow distributions (28). The terminal resistance (R_t_ = R_c_ + R_d_) was then calculated from mean BP and PC-MRI flow measurements and distributed between the remaining resistance parameters by adjusting R_c_ to R_t_ ratios (6–10%) for each outlet using the pulse pressure method, (29, 30) thereby replicating measured BP. An augmented-Lagrangian formulation (31) for constraining velocity profiles at model outlets was used to mitigate instabilities often occurring during flow deceleration and diastole. Vessel deformability (32) was included in FSI simulations as discussed elsewhere (15). Briefly, a wall thickness of 0.15 cm (33) was implemented from literature for all models and the Young's modulus was then adjusted iteratively until the AscAo mean luminal displacement was within 5% of the values obtained from PC-MRI magnitude measurements.

### Computational Simulations

Simulations were performed using a novel stabilized finite element method to solve the conservation of mass (continuity), balance of fluid momentum (Navier-Stokes), and vessel wall elastodynamics equations (32). Simulations were run for 4–6 cardiac cycles until the flow rate and BP fields yielded periodic solutions.

Blood flow velocity, BP, and wall displacement were visualized using ParaView (Kitware, Clifton Park, NY, USA). TAWSS (34) and the oscillatory shear index (OSI) (25) was then calculated as previously described. Low TAWSS is generally thought to promote atherogenesis, as is elevated OSI, an index of directional changes in WSS. Low OSI indicates WSS is unidirectional, while a value of 0.5 is indicative of bidirectional WSS with a time-average value of zero. These indices were quantified in several ways. Values for TAWSS and OSI were extracted longitudinally along the inner and outer curvatures of the thoracic aorta, as well as along its anatomic right, and left sides as described previously (14, 35). This was done because prior imaging studies found that local values for these indices were statistically different from circumferential averages (36), thereby motivating the need to report detailed local WSS maps in computational studies. To visualize these indices locally, the surface of each vessel was unwrapped and mapped into a θ*, l* rectangular domain, where θ and *l* are the circumferential and longitudinal coordinates of each point on the vessel wall (37). Data presented represent an average of nearest-neighbor values in 2% increments along the length of the aorta. This distance was made consistent between patients and control participants using dimensional information from imaging data, and then normalized from 0 to 1. TAWSS values from 0 to 50 dyn/cm^2^ are particularly interesting from the perspective of the vascular response to hemodynamics and are also highlighted in histograms (2 dyn/cm^2^ bins) for values within this range. Histograms are also provided for the full range of OSI, 0–0.5 (0.2 unit bins) (22, 38).

## Results

**Figure 1:**
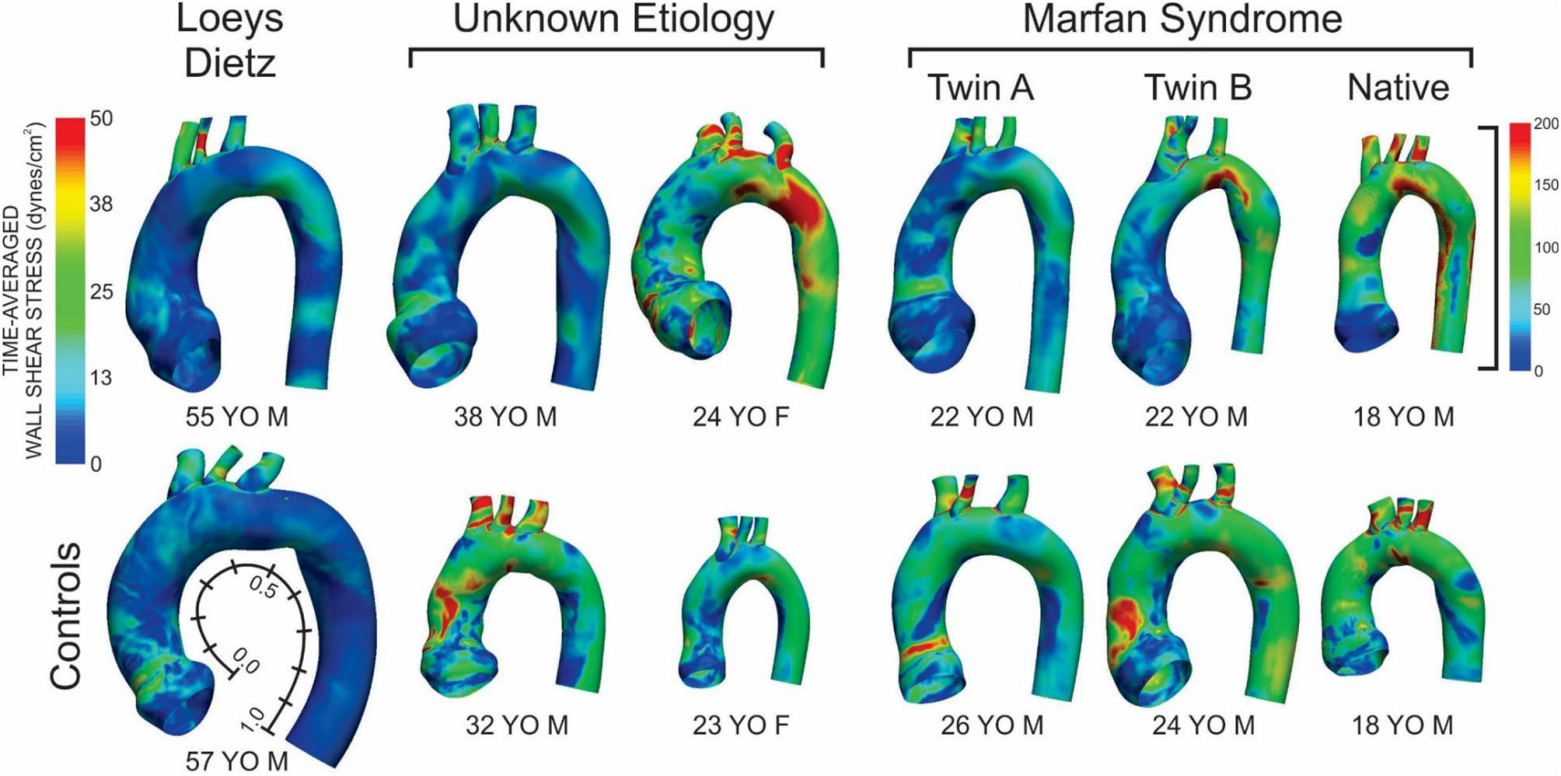
Time-averaged wall shear stress (TAWSS) distributions for the six patients with the various connective tissue diseases studied (top row) and age- and gender-matched control participants to which each was compared (bottom row). The size of the models displaying the TAWSS results shown is relative to each other using the descending aortic outlet dimensions. Data in Figures 2, 4 are presented along the length of the aorta. This distance was made consistent between patients and control participants using dimensional information from imaging data and then normalized from 0 to 1 as shown beside the model and TAWSS from the leftmost control participant.

Fluid structure interaction simulations for all patients, and their respective controls, yielded the TAWSS and OSI results shown in Figures 1, 3, respectively. The size of the models shown is relative to each other using the descending aortic outlet dimension as reference. A summary of dimensions following model construction is also provided in Table 1. In nearly all cases, the AscAo dimension was greater in patients than the respective control participant. Conversely, with the exception of the female patient having connective tissue disease of unknown etiology, the dAo dimension was of smaller caliber compared to the age- and gender-matched control participant. The ratio of AscAo to dAo for patients was consequently 10.6–29.3% larger than AscAo:dAo ratio for control participants.

### Patient With Loeys-Dietz Disease

**Figure 2:**
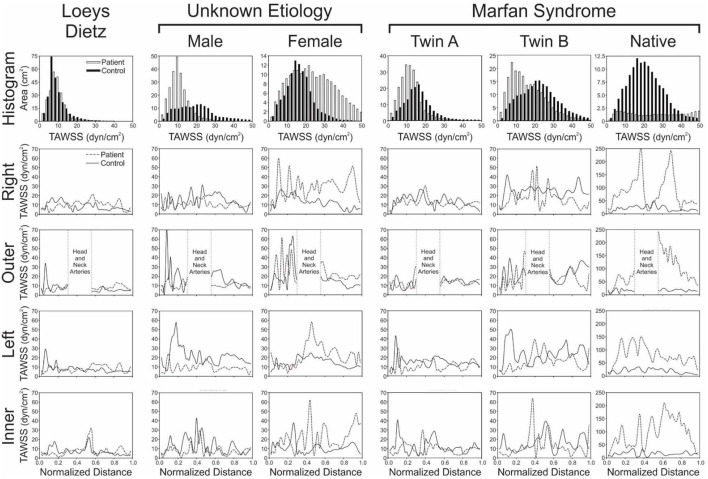
Local quantification of TAWSS results. The top row shows histograms (2 dyn/cm^2^ bins) of the area (cm^2^) from each model surface exposed to TAWSS values from 0 to 50 dyn/cm^2^ (connective tissue disease = solid bars; age- and gender-matched controls = hollow bars). Longitudinal TAWSS distributions along the outer, anatomic right, anatomic left, and inner curvatures of the aorta are also shown in subsequent rows for the patients with various connective tissue disease (dashed lines) vs. their associated age- and gender-matched controls (solid lines). Low TAWSS is generally thought to promote atherogenesis, so skewing of histogram results toward lower values could be interpreted as less ideal, as could overall lower values of TAWSS along the aortic surfaces.

The patient with Loeys-Dietz disease and corresponding control had the lowest overall distributions of TAWSS due primarily to the large AscAo dimensions in the setting of normal range cardiac output values (e.g., Loeys-Dietz = 3.0 L/min/m^2^; control = 2.5 L/min/m^2^). The histogram of TAWSS values shown in Figure 2 reveals the similarity in distributions between the Loeys-Dietz and control models. The smaller caliber of the dAo relative to the AscAo in the patient with Loeys-Dietz disease relative to its control (mentioned above) also results in slightly higher distributions of TAWSS along the last half of the thoracic aorta quantified (distances from ~0.5 to 1.0).

**Figure 3:**
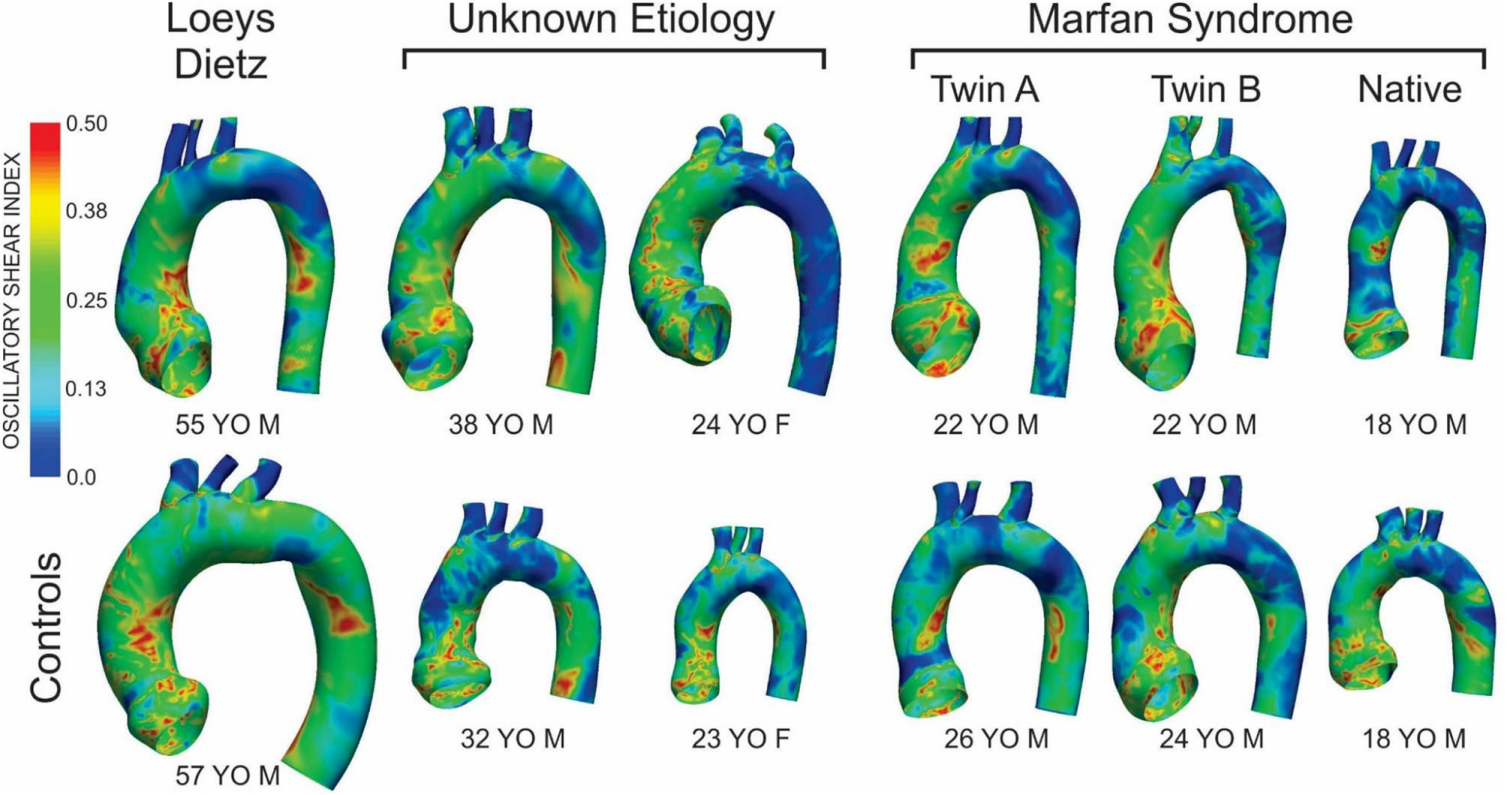
Distributions of oscillatory shear index (OSI) for the six patients with the various connective tissue diseases studied (top row) and age- and gender-matched control participants to which each was compared (bottom row). The size of the models displaying the OSI results shown is relative to each other using the descending aortic outlet dimensions.

**Figure 4:**
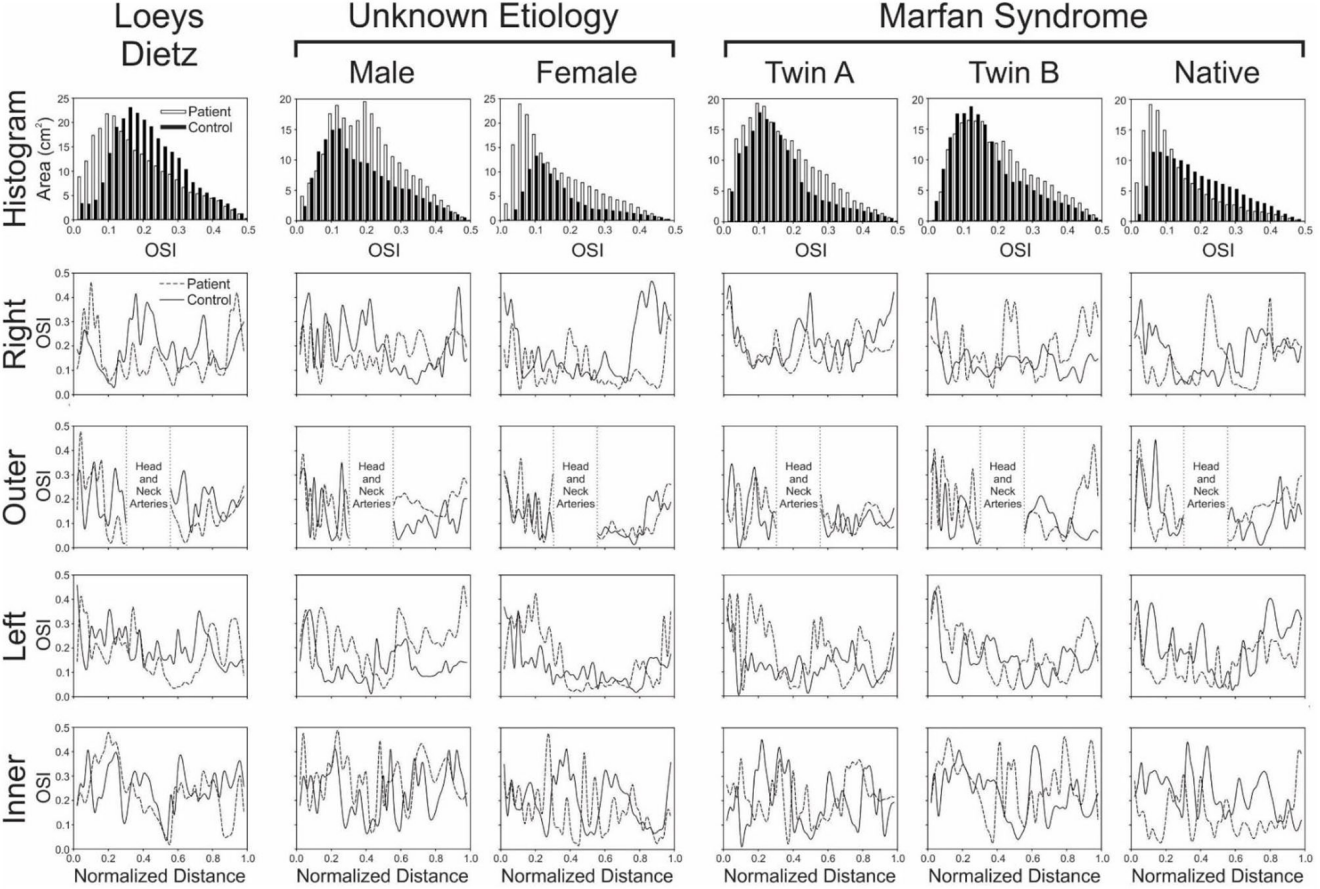
Local quantification of oscillatory shear index (OSI) results. The top row shows histograms (0.2 unit bins) of the area (cm^2^) from each model surface exposed to OSI values from 0 to 0.5 (connective tissue disease = solid bars; age- and gender-matched controls = hollow bars). Longitudinal OSI distributions along the outer, anatomic right, anatomic left, and inner curvatures of the aorta are also shown in subsequent rows for the patients with various connective tissue disease (dashed lines) vs. their associated age- and gender-matched controls (solid lines). Higher OSI values are generally thought to promote atherogenesis, so skewing of histogram results toward higher values could be interpreted as less ideal, as could overall higher values of OSI along the outer, anatomic right, anatomic left, and inner curvatures of the aorta.

There were modest differences in OSI for the patient with Loeys-Dietz disease relative to the control participant (Figure 3). The histogram (Figure 4) shows similar amounts of elevated OSI values >0.4 for both models. The histogram from the patient with Loeys-Dietz disease showed that less of the luminal surface was exposed to elevated OSI (0.2–0.4) shown to be atherogenic in prior reports. The difference in OSI values within this region corresponded to the transverse arch of the control patient (Figure 3), which also seems to have slight out-of-plane morphology in this region compared to the more traditional arch of the patient with Loeys-Dietz disease. These differences can also be seen in the longitudinal plots (e.g., right, outer, and left surfaces at distances of ~0.4–0.6).

### Patients With Connective Tissue Disease of Unknown Etiology

The TAWSS histogram for the male patient with connective tissue disease of unknown etiology reveals a greater portion of the aorta and its branches were exposed to lower TAWSS, particularly in the ascending aorta, relative to control (Figure 2). This is clearly shown in longitudinal TAWSS plots along the outer and left luminal surfaces. Vascular dimensions and morphology were similar for both models, suggesting that these differences may primarily be due to a higher cardiac index for the control participant (e.g., 3.1 L/min/m^2^ for the patient with connective tissue disease vs. 4.5 L/min/m^2^ vs. for the control).

The ascending aorta of the male patient with connective tissue disease of unknown etiology was exposed to a greater area of elevated OSI values in the range of ~0.1–0.4 (Figure 3 and histogram of Figure 4), which occurred along nearly the full length of the left luminal surface and lasted 50–70% of the distance for the right and outer luminal surfaces (Figure 4).

The female patient with connective tissue disease of unknown etiology experienced higher TAWSS along the transverse arch and dAo (Figure 1). This is reflected in all longitudinal plots (Figure 2). This finding appears to correspond to a larger AscAo/dAo ratio for the patient compared to the control.

The larger caliber ascending aorta and its branches for the female patient with connective tissue disease of unknown etiology resulted in a greater area of potentially deleterious OSI values in this region (Figure 3) and more area exposed to the full range of OSI compared to the corresponding control participant. Elevated OSI values were most pronounced along the left luminal surface (Figure 4).

### Patients With Marfan Syndrome

TAWSS for the native (i.e., uncorrected) patient with Marfan syndrome was extremely high relative to the corresponding control participant primarily due to a cardiac index exceeding normal conditions (6.0 L/min/m^2^ vs. 3.2 L/min/m^2^ for the control). The distributions of OSI values were somewhat similar for values > 0.2. By qualitative assessment, the AscAo of the native patient with Marfan syndrome had a greater area exposed to lower OSI (Figure 3), primarily along the inner and left luminal surfaces (Figure 4). Of note, the native patient with Marfan syndrome had the highest Reynolds number of all patients or participants studied (Table 1), and the value suggests flow was not laminar in the aorta of this patient, likely leading to spatial differences in OSI within the AscAo.

The twin patients with Marfan syndrome, who previously underwent aortic root replacement, had similar TAWSS distributions. The computational model labeled as Twin B for this study had a more dilated proximal descending aorta in the vicinity of the ductus ligamentum, which impacted indices of WSS. Differences between the computational models of each twin and their respective control model were mainly a function of cardiac index. TAWSS was generally lower in the dAo of both twins after surgery compared to their respective controls (Figure 1). This was most pronounced along the left luminal surface of the AscAo (Figure 2).

Distributions (Figure 3) and histograms (Figure 4) of OSI values for the twin patients with Marfan syndrome, who previously underwent aortic root replacement, were also similar with morphological differences in the vicinity of the ductus ligamentum mostly impacting the luminal surface at a normalized distance of ~0.4–0.6. This area was not replaced in either subject and reflects their native aortic properties.

## Discussion

Blood flow patterns in the aortic arch and dAo are unique compared with other portions of the arterial vasculature. This is evident when considering the Reynolds number, a dimensionless index used to characterize fluid flow (2). Specifically, Reynolds number describes the ratio of convective inertial forces to viscous forces. In general, Reynolds number values <2,200 constitute laminar flow where adjacent layers of fluid move in layers without mixing, while those >2,200 may be characterized as transitional or turbulent depending on specific details of the local flow domain. Under normal conditions, the thoracic aorta experiences Reynolds numbers on the order of 1,500 (mean) as a result of its large caliber and high blood flow rates. These Reynolds number values indicate blood flow is generally laminar throughout the cardiac cycle, but there are undoubtedly portions of the cardiac cycle during which blood flow transiently becomes transitional and/or turbulent. Characterizing any deviations from these normal flow patterns may be important for optimal operative interventions for the thoracic aorta with the aim being to decrease the likelihood of later, long-term aortic pathology. For example, transient periods of turbulence during systole (due to modest differences in local vessel geometry from thoracic aortic diseases or surgery) could cause downstream disturbances.

This investigation builds upon prior work by conducting FSI, in contrast to rigid CFD, with modeling that includes local wall deformation of the aorta for patients with Loeys-Dietz, connective tissue disease of unclear etiology, and native (i.e., untreated) Marfan syndrome, as well as twin patients with Marfan syndrome who underwent valve sparing aortic root repair. We matched changes in dimension by PC-MRI measurements by assigning local tissue properties and iterating Young's modulus until the deformation matched that observed *in vivo*. Physiological inflow profiles are uniquely implemented by imposing a restricted cross-sectional flow determined from time-varying PC-MRI magnitude data at the level of the valve (21) and outlet boundary conditions for PC-MRI data are added to reflect clinical measurements (14, 15). Our goal with these model improvements was to more accurately replicate the physiological conditions for patients with these connective tissue anomalies, and therefore reveal differences in WSS and OSI vs. age- and gender-matched control participants in a pilot study.

In nearly all cases, the AscAo dimension was greater in patients with connective tissues diseases relative to their respective control. With the exception of the female patient with connective tissue disease of unknown etiology, the dAo dimension was of smaller caliber when compared to the corresponding control. Differences in TAWSS and OSI were driven by these local morphological differences and cardiac output. Unfortunately, it is difficult to speculate on the cause of smaller dAo dimensions in most patients relative to controls given the small sample size and heterogeneity. This finding should be validated in larger studies with groups of patients having similar genetic anomalies.

A unique presentation of the results of this study centers on the twin patients with Marfan syndrome who previously underwent aortic root replacement. The computational model labeled as Twin B for this study had a more pronounced proximal descending aorta in the vicinity of the ductus ligamentum that impacted indices of WSS relative to Twin A. As with the other patients studied here, differences between the computational results of each twin and their respective control were mainly a function of cardiac index. Nonetheless, TAWSS was generally lower in the AscAo of both twins after surgery compared to their respective controls (Figure 1). Interpretation of these findings prompted review of literature that may be applicable. With twin gestation, there is a higher incidence of congenital heart disease. For monozygotic twins (65% with one chorion), the incidence of congenital heart malformations may be six times that for a singleton. For monozygotic twins, twin-twin transfusion syndrome may play a role in the development of CHD (39). The twins in our cohort were fraternal twins (i.e., dichorionic) inheriting the gene mutation for Marfan syndrome. Marfan syndrome is almost exclusively inherited in an autosomal-dominant manner, although rare case reports have described recessive fibrillin 1 gene (FBN1) mutations (40). For these dichorionic twins, twin-twin transfusion does not explain the CHD, but rather genetic inheritance explains their clinical course. In other dichorionic twins with congenital heart diseases such a coarctation of the aorta, altered fetal-placental hemodynamics sometimes resulting from fetal growth restriction can contribute to the development of their congenital heart disease (41).

The results of this study build from and extend existing simulation results for these patient populations. For example, previous work has independently quantified stress and strain fields (42) for three patients with Marfan syndrome before and after surgery to implement personalized external aortic root support, as well as aortic flow patterns and resulting distributions of WSS using imposed flow and pressure waveforms allowing for qualitative comparison of velocity patterns to PC-MRI (20). Although the fluid flow version of the study used rigid wall CFD, the results are generally consistent with this study in that models created from data before personalized external aortic root support had larger AscAo diameters, leading to more flow disturbances (20). More recent work reported the ratio of circumferential to longitudinal WSS in idealized models informed by the data of patient with Marfan syndrome with stable or dilating aneurysms (43). To the best of our knowledge, there is a paucity of studies characterizing altered blood flow patterns for patients with connective tissue anomalies beyond Marfan syndrome. Despite the scarcity of such studies, there is a 2015 report that quantified aortic dimensions and indices of aortic stiffness in patients with connective tissue disorders using MRI (8). While the finding of elevated stiffness in patients with connective tissue disorders from this study suggests that rigid CFD models may be appropriate for this patient population, it also points to the importance of accurately replicating deformation in patients with connective tissue disorders, who may not yet have experienced an increase in stiffness and associated adverse changes in WSS indices.

The results of this study should be considered relative to several potential limitations. Our investigation studied alterations in WSS indices locally in the proximal thoracic aorta and its branches given the hallmark capacitive function for this region of the arterial vasculature. There is evidence, however, that connective tissue disorders such as Marfan syndrome may also impact central aortic flow dynamics by virtue of altered distal resistance vessels (44). Although our outlet boundary conditions do account for downstream vascular resistance, the impact of changes in resistance vessel due to each connective tissue disease was not explicitly included in the outflow boundary conditions imposed for this study. To date, it is not known which specific, or combination of, WSS indices are directly linked to vascular pathologies for patients with connective tissue diseases. The influence of each index likely also depends on the patient population and its predominant pathology (e.g., stiffening, neointimal hyperplasia, and aneurysm rupture) (4, 45, 46). The small sample size for each connective tissue disease does undoubtedly present a limitation to extrapolating the results to the full population of patients with Loeys-Dietz, patients with connective tissue disease of unclear etiology, and those with Marfan syndrome (native and status post valve sparing root replacement). The cardiac index for the native (i.e., uncorrected) patient with Marfan syndrome was higher than expected based on elevated heart rate and hyperdynamic left ventricular ejection fraction. It is also possible that this patient had some anxiety during the MRI session. The goal of this study was to remain patient specific in terms of boundary conditions. However, when considering the results of this study, it may be interesting to conduct an idealized parameter-based study to quantify the impact of morphology and cardiac output independently. Such work is planned for the future. The Lagrangian multiplier approach implemented for constraining outlet velocity profiles can be replaced in future work by a backflow stabilization treatment that is thought to be less intrusive to the flow field, computationally inexpensive, and has been implemented in Simvascular (47). While this study did impose a plug velocity profile at model inlets, it was restricted by the time-varying cross section determined from PC-MRI using novel methods previously developed in our lab (21). Upon implementation of these methods, we quantified the impact of valve morphology on aortic hemodynamics and identified regions most influenced by the inlet, including the ascending aorta that has previously been a site of dilation for patients with Marfan syndrome as a result of local flow patterns. Besides this approach, computational studies of the thoracic aorta to date have typically introduced blood flow in one of two ways. In one approach, PC-MRI is used to temporally sample the velocity profile downstream of the valve and input this measured profile directly into the model. While not directly including the valve, its impact can be manifested in the data that is obtained, but this requires appropriate through- and in-plane velocity encoding to adequately resolve flow features being input into the CFD model. This approach may be difficult to implement within the constraints of a clinical setting as it can require specialized sequences not routinely implemented and obtains data that are more detailed than those commonly used in clinical diagnosis. One alternative approach has been to construct CFD models with their inlet beginning just distal to the aortic sinuses, impose the blood flow waveform measured downstream as an assumed velocity profile at the model inlet, and allow the curvature of the arch to influence resulting flow patterns (14). While this technique does not use the complete spatial velocity information, it does not require specialized sequences, minimizes the introduction of noise at the model inflow due to inadequate velocity encoding, and allows for improved temporal resolution compared to 3-component PC-MRI (48). The methods of Wendell et al. used in this study (21) allow for more accurate representation of the impact of the aortic valve on computational studies of the thoracic aorta while still using data obtained as part of a routine clinical imaging session. The patients with connective tissue disorders of unknown etiology were suspected to have hereditary (or genetic) aortopathy, but negative testing for known genetic variants. The yield for current gene panels for thoracic aortic disease is only ~30%, even in patients with high clinical suspicion. Hence, unfortunately, it is a common situation in our clinic to have suspected genetic etiology but a negative gene panel. This may be interpreted as not yet identifying the applicable gene(s) in that individual/family. We often pursue whole-exome sequencing in such patients, but even then the results often do not identify a causative genetic variant. This is a limitation that we are working to mitigate in the future as having complete genetic data for future cohorts would greatly enhance our understanding of the results presented for larger populations of patients.

## Conclusion

The methods employed represent some of the most advanced vascular modeling tools available such as deformable walls, dynamically varying valvular area at the inlet of the model, physiological boundary conditions, and the use of age- and gender-matched controls. Despite some potential limitations outlined above in implementing these tools, the lack of computational modeling data for those patients with connective tissue diseases makes the current pilot data interesting and relevant. We are optimistic that the results of this study can serve as a foundation for larger future studies with the connective tissue disorders presented here.

## Data Availability Statement

The datasets presented in this article are not readily available because raw data supporting the conclusions of this article can only be available by the authors to the extent possible by the institutional approvals governing the research presented. Requests to access the datasets should be directed to jladisa@mcw.edu.

## Ethics Statement

Ethical review and approval was obtained for the study using data from human participants in accordance with local legislation and institutional requirements. The patients/participants provided their written informed consent to participate in this study.

## Author Contributions

JAC: patient recruitment clinical expertise for manuscript development/review. HW and RD: computational simulations, analysis/interpretation of computational results, and approval of the article. MS and JRC: patient recruitment, supervision of MRI scanning, and clinical expertise for manuscript development/review. JL: concept/design, methodological developments, analysis/interpretation of computational results, drafting article, and approval of article. All authors contributed to the article and approved the submitted version.

## Funding

Funding was provided through internal grant funding at the Medical College of Wisconsin (to MS) and an Educational Research Agreement between Children's Hospital of Wisconsin and Marquette University.

## Conflict of Interest

The authors declare that the research was conducted in the absence of any commercial or financial relationships that could be construed as a potential conflict of interest.

## Publisher's Note

All claims expressed in this article are solely those of the authors and do not necessarily represent those of their affiliated organizations, or those of the publisher, the editors and the reviewers. Any product that may be evaluated in this article, or claim that may be made by its manufacturer, is not guaranteed or endorsed by the publisher.

## References

[1] Liu X Sun A Fan Y Deng X. Physiological significance of helical flow in the arterial system and its potential clinical applications. Ann Biomed Eng. (2015) 43:3–15. 10.1007/s10439-014-1097-2 25169424

[2] Westerhof N Stergiopulos N Noble MIM. Snapshots of hemodynamics an aid for clinical research and graduate education. New York: Springer (2005).

[3] Dobrin PB. Poststenotic dilatation. Surg Gynecol Obstet. (1991) 172:503–8. 2035144

[4] Wentzel JJ Corti R Fayad ZA Wisdom P Macaluso F Winkelman MO. Does shear stress modulate both plaque progression and regression in the thoracic aorta? Human study using serial magnetic resonance imaging. J Am Coll Cardiol. (2005) 45:846–54. 10.1016/j.jacc.2004.12.026 15766817

[5] Hathcock JJ. Flow effects on coagulation and thrombosis. Arterioscler Thromb Vasc Biol. (2006) 26:1729–37. 10.1161/01.ATV.0000229658.76797.30 16741150

[6] Canadas V Vilacosta I Bruna I Fuster V. Marfan syndrome. Part 1: pathophysiology and diagnosis. Nat Rev Cardiol. (2010) 7:256–65. 10.1038/nrcardio.2010.30 20351703

[7] Canadas V Vilacosta I Bruna I Fuster V. Marfan syndrome. Part 2: treatment and management of patients. Nat Rev Cardiol. (2010) 7:266–76. 10.1038/nrcardio.2010.31 20351702

[8] Prakash A Adlakha H Rabideau N Hass CJ Morris SA Geva T. Segmental aortic stiffness in children and young adults with connective tissue disorders: relationships with age, aortic size, rate of dilation, and surgical root replacement. Circulation. (2015) 132:595–602. 10.1161/CIRCULATIONAHA.114.014934 26115544

[9] Coogan JS Chan FP Ladisa JF Jr Taylor CA Hanley FL Feinstein JA. Computational fluid dynamic simulations for determination of ventricular workload in aortic arch obstructions. J Thorac Cardiovasc Surg. (2013) 145:489–95.e1. 10.1016/j.jtcvs.2012.03.051 22516390

[10] Figueroa CA Taylor CA Yeh V Chiou AJ Gorrepati ML Zarins CK. Preliminary 3D computational analysis of the relationship between aortic displacement force and direction of endograft movement. J Vasc Surg. (2010) 51:1488–97. 10.1016/j.jvs.2010.01.058 20488325 PMC2874723

[11] Kim HJ Vignon-Clementel IE Figueroa CA LaDisa JF Jansen KE Feinstein JA. On coupling a lumped parameter heart model and a three-dimensional finite element aorta model. Ann Biomed Eng. (2009) 37:2153–69. 10.1007/s10439-009-9760-8 19609676

[12] Kwon S Feinstein JA Dholakia RJ LaDisa JF Jr. Quantification of local hemodynamic alterations caused by virtual implantation of three commercially available stents for the treatment of aortic coarctation. Pediatr Cardiol. (2014) 35:732–40. 10.1007/s00246-013-0845-7 24259013 PMC3959287

[13] LaDisa JF Jr Bowers M Harmann L Prost R Doppalapudi AV Mohyuddin T. Time-efficient patient-specific quantification of regional carotid artery fluid dynamics and spatial correlation with plaque burden. Medical physics. (2010) 37:784–92. 10.1118/1.3292631 20229888 PMC2826384

[14] LaDisa JF Jr Dholakia RJ Figueroa CA Vignon-Clementel IE Chan FP Samyn MM. Computational simulations demonstrate altered wall shear stress in aortic coarctation patients treated by resection with end-to-end anastomosis. Congenit Heart Dis. (2011) 6:432–43. 10.1111/j.1747-0803.2011.00553.x 21801315 PMC3208403

[15] LaDisa JF Jr Figueroa CA Vignon-Clementel IE Kim HJ Xiao N Ellwein LM. Computational simulations for aortic coarctation: representative results from a sampling of patients. J Biomech Eng. (2011) 133:091008. 10.1115/1.4004996 22010743 PMC3705983

[16] Les AS Shadden SC Figueroa CA Park JM Tedesco MM Herfkens RJ. Quantification of hemodynamics in abdominal aortic aneurysms during rest and exercise using magnetic resonance imaging and computational fluid dynamics. Ann Biomed Eng. (2010) 38:1288–313. 10.1007/s10439-010-9949-x 20143263 PMC6203348

[17] Marsden AL Bernstein AJ Reddy VM Shadden SC Spilker RL Chan FP. Evaluation of a novel Y-shaped extracardiac Fontan baffle using computational fluid dynamics. J Thorac Cardiovasc Surg. (2009) 137:394–403.e2. 10.1016/j.jtcvs.2008.06.043 19185159

[18] Tang BT Fonte TA Chan FP Tsao PS Feinstein JA Taylor CA. Three-dimensional hemodynamics in the human pulmonary arteries under resting and exercise conditions. Ann Biomed Eng. (2011) 39:347–58. 10.1007/s10439-010-0124-1 20640512

[19] Vignon-Clementel IE Figueroa CA Jansen KE Taylor CA. Outflow boundary conditions for 3D simulations of non-periodic blood flow and pressure fields in deformable arteries. Comput Methods Biomech Biomed Engin. (2010) 13:625–40. 10.1080/10255840903413565 20140798

[20] Singh SD Xu XY Wood NB Pepper JR Izgi C Treasure T. Aortic flow patterns before and after personalised external aortic root support implantation in Marfan patients. J Biomech. (2016) 49:100–11. 10.1016/j.jbiomech.2015.11.040 26654673

[21] Wendell DC Samyn MM Cava JR Ellwein LM Krolikowski MM Gandy KL. Including aortic valve morphology in computational fluid dynamics simulations: initial findings and application to aortic coarctation. Med Eng Phys. (2013) 35:723–35. 10.1016/j.medengphy.2012.07.015 22917990 PMC3577975

[22] Wendell DC Samyn MM Cava JR Krolikowski MM LaDisa JF Jr. The impact of cardiac motion on aortic valve flow used in computational simulations of the thoracic aorta. J Biomech Eng. (2016) 138:0910011–111. 10.1115/1.4033964 27367143 PMC4967883

[23] Sahni O Muller J Jansen KE Shephard MS Taylor CA. Efficient anisotropic adaptive discretization of the cardiovascular system. Comput Methods Biomech Biomed Engin. (2006) 195:5634–55. 10.1016/j.cma.2005.10.01819727874

[24] Muller J Sahni O Li X Jansen KE Shephard MS Taylor CA. Anisotropic adaptive finite element method for modeling blood flow. Comput Methods Biomech Biomed Engin. (2005) 8:295–305. 10.1080/10255840500264742 16298851

[25] Tang BT Cheng CP Draney MT Wilson NM Tsao PS Herfkens RJ. Abdominal aortic hemodynamics in young healthy adults at rest and during lower limb exercise: quantification using image-based computer modeling. Am J Physiol Heart Circ Physiol. (2006) 291:H668–76. 10.1152/ajpheart.01301.2005 16603687

[26] Vignon-Clementel IE Figueroa CA Jansen KE Taylor CA. Outflow boundary conditions for three-dimensional finite element modeling of blood flow and pressure in arteries. Comput Methods Appl Mech Eng. (2006) 195:3776–96. 10.1016/j.cma.2005.04.014

[27] Laskey WK Parker HG Ferrari VA Kussmaul WG Noordergraaf A. Estimation of total systemic arterial compliance in humans. J Appl Physiol. (1990) 69:112–9. 10.1152/jappl.1990.69.1.112 2394640

[28] Stergiopulos N Young DF Rogge TR. Computer simulation of arterial flow with applications to arterial and aortic stenoses. J Biomech. (1992) 25:1477–88. 10.1016/0021-9290(92)90060-E 1491023

[29] O'Rourke MF Safar ME. Relationship between aortic stiffening and microvascular disease in brain and kidney: cause and logic of therapy. Hypertension. (2005) 46:200–4. 10.1161/01.HYP.0000168052.00426.65 15911742

[30] Stergiopulos N Segers P Westerhof N. Use of pulse pressure method for estimating total arterial compliance *in vivo*. Am J Physiol Heart Circ Physiol. (1999) 276:H424–8. 10.1152/ajpheart.1999.276.2.H424 9950841

[31] Kim HJ Figueroa CA Hughes TJR Jansen KE Taylor CA. Augmented Lagrangian method for constraining the shape of velocity profiles at outlet boundaries for three-dimensional Finite Element simulations of blood flow. Comput Methods Appl Mech Eng. (2009) 198:3551–66. 10.1016/j.cma.2009.02.012

[32] Figueroa CA Vignon-Clementel IE Jansen KE Hughes TJR Taylor CA. A coupled momentum method for modeling blood flow in three-dimensional deformable arteries. Comput Methods Appl Mech Eng. (2006) 195:5685–706. 10.1016/j.cma.2005.11.011

[33] Westerhof N Bosman F De Vries CJ Noordergraaf A. Analog studies of the human systemic arterial tree. J Biomech. (1969) 2:121–43. 10.1016/0021-9290(69)90024-4 16335097

[34] Eslami P Tran J Jin Z Karady J Sotoodeh R Lu MT. Effect of wall elasticity on hemodynamics and wall shear stress in patient-specific simulations in the coronary arteries. J Biomech Eng. (2020) 142: 0245031–310. 10.1115/1.4043722 31074768 PMC7105147

[35] Samyn MM Dholakia R Wang H Co-Vu J Yan K Widlansky ME. Cardiovascular magnetic resonance imaging-based computational fluid dynamics/fluid-structure interaction pilot study to detect early vascular changes in pediatric patients with type 1 diabetes. Pediatr Cardiol. (2015) 36:851–61. 10.1007/s00246-014-1071-7 25577225

[36] Frydrychowicz A Stalder AF Russe MF Bock J Bauer S Harloff A. Three-dimensional analysis of segmental wall shear stress in the aorta by flow-sensitive four-dimensional-MRI. J Magn Reson Imaging. (2009) 30:77–84. 10.1002/jmri.21790 19557849

[37] Gundert TJ Shadden SC Williams AR Koo BK Feinstein JA Ladisa JF Jr. A rapid and computationally inexpensive method to virtually implant current and next-generation stents into subject-specific computational fluid dynamics models. Ann Biomed Eng. (2011) 39:1423–37. 10.1007/s10439-010-0238-5 21203844

[38] Ellwein L. M SM M. D Schindler-Ivens S Liebham S LaDisa JF Jr. Toward translating near-infrared spectroscopy oxygen saturation data for the noninvasive prediction of spatial and temporal hemodynamics during exercise. Biomech Model Mechanobiol. (2017) 16:75–96. 10.1007/s10237-016-0803-4 27376865 PMC5217163

[39] Balasubramanian R Vuppalapati S Avanthika C Jhaveri S Peddi NC Ahmed S. Epidemiology, genetics and epigenetics of congenital heart diseases in twins. Cureus. (2021) 13:e17253. 10.7759/cureus.17253 34540478 PMC8448266

[40] Hilhorst-Hofstee Y Rijlaarsdam ME Scholte AJ Swart-van den Berg M Versteegh MI van der Schoot-van Velzen I. The clinical spectrum of missense mutations of the first aspartic acid of cbEGF-like domains in fibrillin-1 including a recessive family. Hum Mutat. (2010) 31:E1915–27. 10.1002/humu.21372 20886638 PMC3051827

[41] Piacentini G Mastromoro G Bottoni A Romano V Riccardi R Orfeo L. Pathophysiology of coarctation of the aorta in dichorionic twins with growth discordance. Ultrasound Obstet Gynecol. (2022) 59:124–5. 10.1002/uog.23717 34159669

[42] Singh SD Xu XY Pepper JR Treasure T Mohiaddin RH. Biomechanical properties of the Marfan's aortic root and ascending aorta before and after personalised external aortic root support surgery. Med Eng Phys. (2015) 37:759–66. 10.1016/j.medengphy.2015.05.010 26054807

[43] Pons R Guala A Rodriguez-Palomares JF Cajas JC Dux-Santoy L Teixido-Tura G. Fluid-structure interaction simulations outperform computational fluid dynamics in the description of thoracic aorta haemodynamics and in the differentiation of progressive dilation in Marfan syndrome patients. R Soc Open Sci. (2020) 7:191752. 10.1098/rsos.191752 32257331 PMC7062053

[44] Syyong HT Chung AW Yang HH van Breemen C. Dysfunction of endothelial and smooth muscle cells in small arteries of a mouse model of Marfan syndrome. Br J Pharmacol. (2009) 158:1597–608. 10.1111/j.1476-5381.2009.00439.x 19814726 PMC2795226

[45] Meng H Tutino VM Xiang J Siddiqui A. High WSS or low WSS? Complex interactions of hemodynamics with intracranial aneurysm initiation, growth, and rupture: toward a unifying hypothesis. AJNR Am J Neuroradiol. (2014) 35:1254–62. 10.3174/ajnr.A3558 23598838 PMC7966576

[46] LaDisa JF Jr Olson LE Molthen RC Hettrick DA Pratt PF Hardel MD. Alterations in wall shear stress predict sites of neointimal hyperplasia after stent implantation in rabbit iliac arteries. Am J Physiol Heart Circ Physiol. (2005) 288:H2465–75. 10.1152/ajpheart.01107.2004 15653759

[47] Moghadam ME Bazilevs Y Hsia TY Vignon-Clementel IE Marsden AL Allianc MCH. Comparison of outlet boundary treatments for prevention of backflow divergence with relevance to blood flow simulations. Comput Mech. (2011) 48:277–91. 10.1007/s00466-011-0599-0

[48] Lotz J Meier C Leppert A Galanski M. Cardiovascular flow measurement with phase-contrast MR imaging: basic facts and implementation. Radiographics. (2002) 22:651–71. 10.1148/radiographics.22.3.g02ma11651 12006694

